# P-1542. Determining *Pseudomonas aeruginosa* antibiotic resistance by leveraging methylation risk scores

**DOI:** 10.1093/ofid/ofae631.1709

**Published:** 2025-01-29

**Authors:** Shuheng Gan, Jiwoong Kim, Juyeon Kim, Andrew E Clark, David E Greenberg, Xiaowei Zhan

**Affiliations:** University of Texas Southwestern Medical Center, Dallas, Texas; University of Texas Southwestern, Dallas, TX; University of Texas Southwestern Medical Center, Dallas, Texas; University of Texas Southwestern Medical Center, Dallas, Texas; UT Southwestern, Dallas, Texas; UTSouthwestern Medical Center, Dallas, Texas

## Abstract

**Background:**

The increasing prevalence of antibiotic-resistant bacteria poses a significant threat to global health, necessitating innovative approaches to predict and manage resistance. Traditional methods for detecting antibiotic resistance primarily rely on phenotypic assays or short-read sequencing techniques, which often overlook the intricate epigenetic modifications that can influence bacterial behavior and resistance mechanisms. One such modification, DNA methylation, has emerged as a critical regulator in bacterial gene expression and pathogenicity. The emergence of long-read sequencing technologies offers a unique opportunity to comprehensively map methylation patterns across bacterial genomes, potentially unveiling novel insights into the mechanisms underlying antibiotic resistance.

**Figure:**
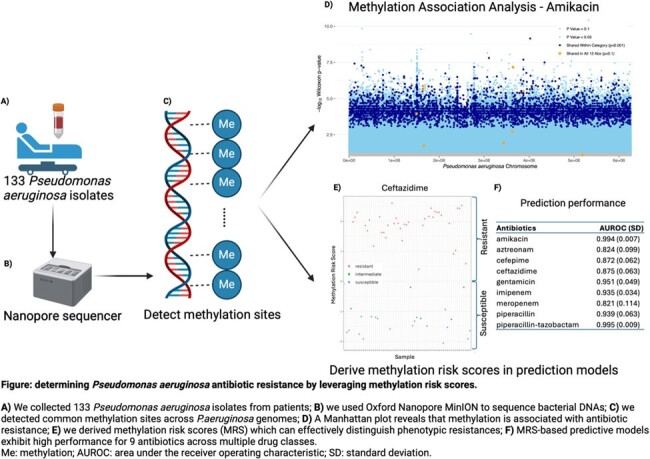

**Methods:**

We systematically sequenced 133

Pseudomonas aeruginosa

bacterial isolates using Oxford nanopore technology and profiled their methylation characteristics.

**Results:**

We detected 3.38 million common methylation sites (average methylation larger than 1%, occurred in more than 10% samples). By applying methylation association analysis, we discovered 10 methylated sites that are statistically associated with resistance to 12 antibiotics. Based on these results, we developed LASSO regression models using novel weighted methylation risk scores. We benchmarked MRSs using cross validation and observed MRS prediction models have high prediction performance for phenotypic resistance to 9 antibiotics across multiple drug classes.

**Conclusion:**

In all, this work demonstrated that long-read derived methylation can serve as a powerful predictor of antibiotic resistance, potentially transforming the field of microbial resistance research.

**Disclosures:**

**David E. Greenberg, MD**, Jannsen: DSMB member|Sarepta Therapeutics: Advisor/Consultant|Shionogi: Grant/Research Support

